# A Case of Transit Internuclear Ophthalmoplegia Following a Motorcycle Accident

**DOI:** 10.31662/jmaj.2022-0215

**Published:** 2023-03-13

**Authors:** Takeya Suzuki, Hidefumi Sano, Takeo Nagura, Mariko Moriya, Junya Tsurukiri

**Affiliations:** 1 Department of Emergency and Critical Care Medicine, Tokyo Medical University Hachioji Medical Center, Tokyo, Japan

**Keywords:** cerebral infarction, head trauma, stroke

A 49-year-old man complained of diplopia after a motorcycle accident. There was no evidence of anisocoria, convergence deficit, ptosis, or motor weakness (Figure 1). Head computed tomography revealed no intracranial hemorrhage (Figure 2). Subsequent magnetic resonance (MR) imaging revealed diffusion restriction on the dorsal pons without T2* shortening and vertebrobasilar artery abnormalities, leading to the diagnosis of internuclear ophthalmoplegia (INO) (Figure 3). The patient denied heredity thrombophilia, collagen disease, or systemic vasculitides. Three days later, the symptoms had spontaneously resolved. A lesion in the medial longitudinal fasciculus caused INO, and major mechanisms of INO are brainstem injury by shear stress due to head blow or secondary brainstem infarction caused by the vertebrobasilar artery perforating branches injury (i.e., compression or kinking) due to shearing force ^(1), (2), (3)^. Without the evidence of hemorrhage, brainstem ischemia was thought to be the possible mechanism in the present case and the factors predisposing to infarction were excluded.

**Figure 1.**
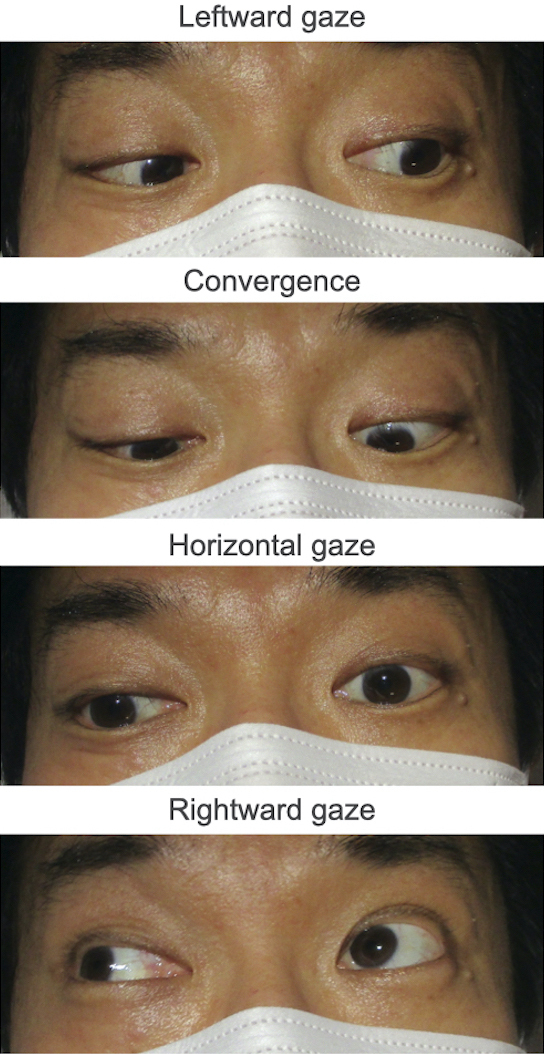


**Figure 2.**
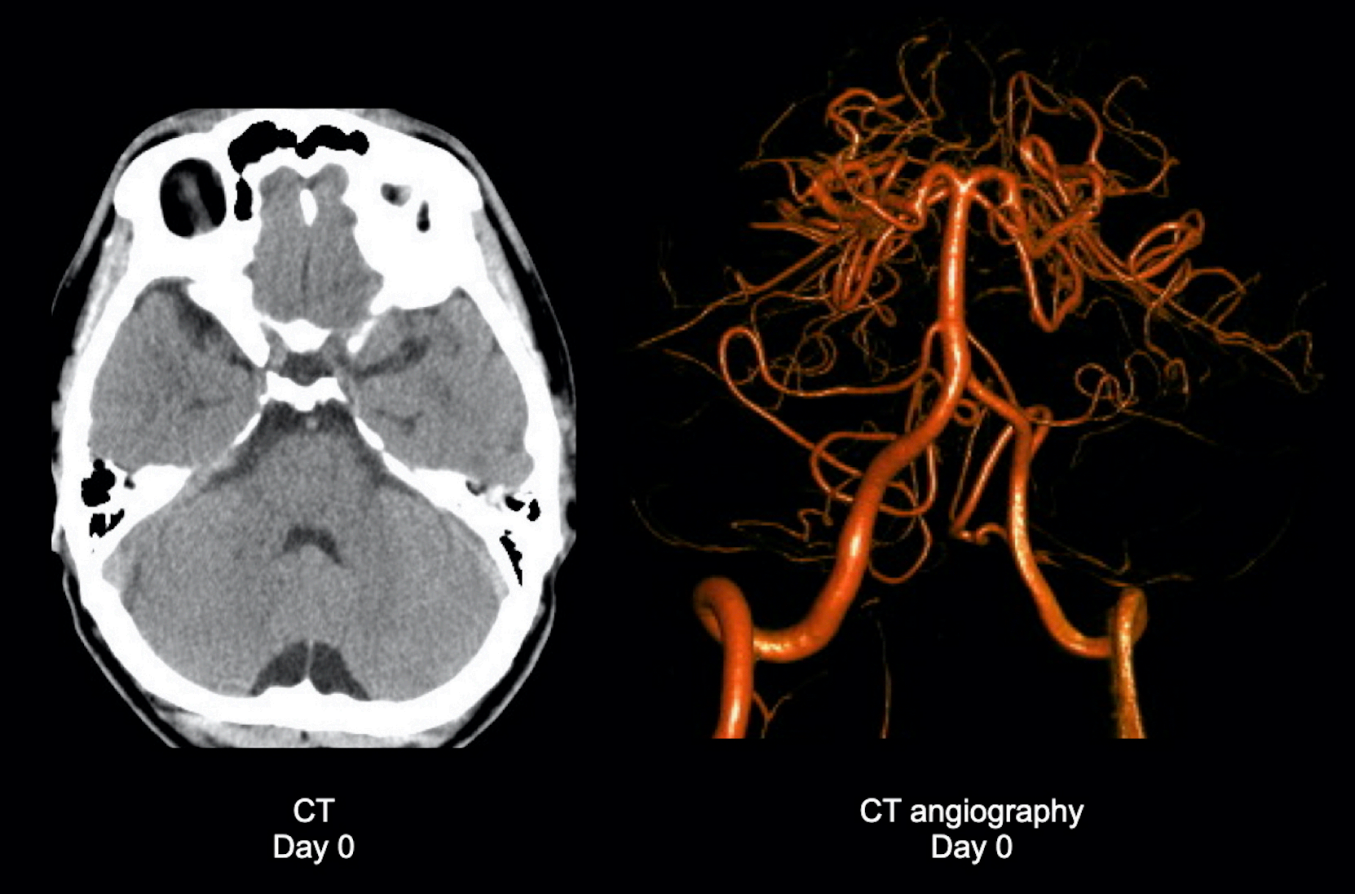


**Figure 3.**
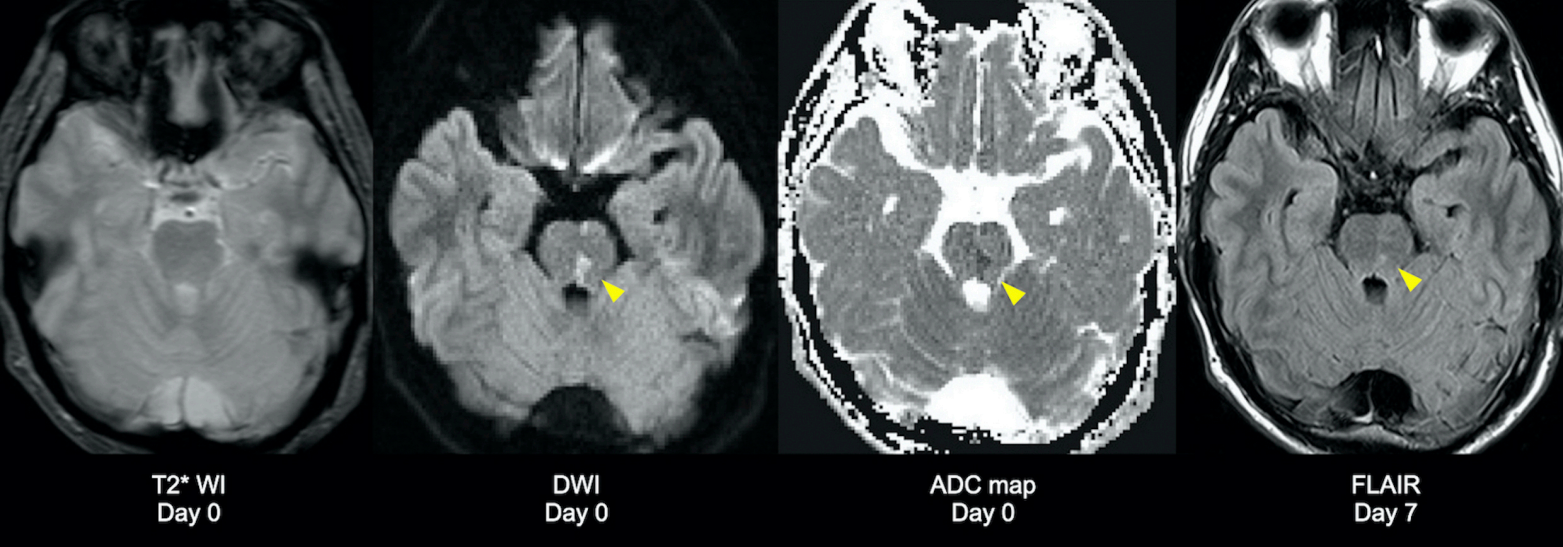


## Article Information

### Conflicts of Interest

None

### Acknowledgement

The authors would like to thank Enago (www.enago.jp) for the English language review.

### Author Contributions

Conceived and designed the experiments: ST, SH

Contributed to interpretation of data: NT, MM

Approved the final version to be submitted: TJ

### Informed Consent

Written informed consent was obtained from the patient for the publication of this case report and any accompanying images.
